# Long Carbon Fibers for Microwave Absorption: Effect of Fiber Length on Absorption Frequency Band

**DOI:** 10.3390/mi11121081

**Published:** 2020-12-06

**Authors:** Hanadi Breiss, Aicha El Assal, Ratiba Benzerga, Chloé Méjean, Ala Sharaiha

**Affiliations:** Univ Rennes, CNRS, IETR—UMR 6164, F-35000 Rennes, France; hanadi.breiss@univ-rennes1.fr (H.B.); aicha.el-assal@univ-rennes1.fr (A.E.A.); chloe.mejean@gmail.com (C.M.); ala.sharaiha@univ-rennes1.fr (A.S.)

**Keywords:** lightweight epoxy foam, carbon fibers, absorbing composite, fiber length, dielectric properties, microwave absorber

## Abstract

This work presents lightweight epoxy foams loaded with very low weight percentages (≤0.5 wt.%) of carbon fibers (CFs) with different lengths (3 mm, 6 mm, and 12 mm) as broadband microwave absorbing materials for anechoic chamber application. The effect of CF length on microwave absorption, especially on the absorption frequency band, is investigated for frequencies between 1 and 15 GHz. For the elaboration of composites, three different methods—spatula, shear mixing, and ultrasounds—are used for the dispersion of CFs. The observation of these CFs, after the dispersion step, shows a high fiber breakage rate when shear mixing is used, unlike when spatula or ultrasounds methods are used. On the other hand, the characterization of the elaborated composites highlights a correlation between the mixing methods, hence the fiber brakeage, and the measured reflection coefficient (reflection loss) of the composites. As a result, the minimum value of the reflection coefficient is shifted toward the high frequencies when the fiber breakage is observed, suggesting that short CFs absorb at high frequencies while long CFs absorb at low frequencies. Dielectric properties, extracted from the measurement in free space, of composites elaborated with different fiber lengths (3 mm, 6 mm, and 12 mm) confirm that short CFs (3 mm) show maximum losses at high frequencies (around 15 GHz) while long CFs (12 mm) show maximum dielectric losses at low frequencies (below 4 GHz). However, no significant variation is observed on the real part of the relative permittivity, as a function of fiber length, for these porous composites loaded with very low CF rates. A hybrid composite, with a mix of different CF lengths, is prepared and characterized. The simulation of the absorption performance of a pyramidal absorber, based on this hybrid composite, is compared to the one of pyramidal absorber based on composites loaded with a single length of carbon fibers. The pyramidal absorber-based hybrid composite predicts the best absorption performance, especially at the low frequency band. The simulated reflection coefficient of this absorber is less than −12 dB in all the studied frequency range, and less than −40 dB for frequencies higher than 3 GHz. This result confirms the interest of using a mix of carbon fiber lengths to achieve a broadband microwave absorber.

## 1. Introduction

The ever-increasing growth in electronic systems in our day-to-day life results a large number of unwanted radiated signals which lead to increased electromagnetic interference (EMI) that is harmful to both the user and nearby electronic equipment [1,2]. These undesirable emissions can be reduced by a proper protection of equipment. In this regard, demands for microwave absorbing materials have increased in order to meet the requirements of the targeted applications; hence, new kinds of microwave absorbing materials have emerged. Beside the lower thickness, lower weight, and higher strength, many studies have focused on finding microwave absorbing material operating over a broad frequency range. For example, in the anechoic chamber, large broadband frequency absorption, commonly between 0.5 and 20 GHz, is required [3,4]. These chambers are covered by lightweight absorbers with various geometries (pyramids, multilayers) and different heights to ensure a total electromagnetic (EM) absorption over a broad frequency band. It should be noted here that the low frequency band (long wavelengths) is the most critical absorption band because it requires a thick absorber, often higher than the quarter wavelength. Currently, the most widely used absorbing materials in the anechoic chambers are the pyramidal absorbers based on polyurethane foam loaded with nanometric carbon particles [3,4,5]. Despite the good absorption performance of these materials, they present some drawbacks, like the volatile charge (nanometric carbon) that induces premature aging of the absorbers and can also be harmful to human health [6]. Moreover, when high pyramidal absorber (height > 1 m) is needed to achieve the absorption at low frequencies, the dipping of these bulky absorbers in the absorbing solution (containing nanometric carbon) becomes difficult, and the immersion amount of the absorbing load cannot be controlled. For the purpose of improving the performance at low frequencies without increasing the height of pyramids, some researches have proposed the incorporation of metallic resonating structures (metamaterials) inside the base of the pyramidal absorbers [7,8]. However, these metallic materials are heavy and exhibit a low corrosion resistance, hence the necessity to find an alternative solution to enhance the absorption at these low frequencies. Recently, different studies focused on new composite material absorbers, especially epoxy composites, owing to their lightweight, corrosion resistance, commercial viability, and ease of processing. This has led to intensive researches with epoxy composites loaded with one or more conductive fillers such as metals [9], carbon materials (carbon black, graphite, graphene, CNT, etc.) [10,11], and metal oxide [12]. The focus has shifted to carbon-based composite materials such as carbon fiber-based composites. These carbon fibers (CFs) have attracted interest as loads in composite materials for several applications thanks to their high strength, high elastic modulus, high corrosion resistance, low density, and low cost [13]. Recently, a lightweight composite material, based on CFs-loaded epoxy foam, was proposed as absorber for the application in anechoic chamber [14]. For example, in the work of Méjean et al., epoxy foam loaded with a low concentration (0.5 wt.%) of CFs has shown a good microwave absorption performance all over the frequency band between 4 and 18 GHz [14]. However, this material still suffers from the bad performance at low frequencies (lower than 4 GHz).

Moreover, the absorption performance of the composite materials depends on their complex permittivity $\left(\varepsilon^{*}\right)$ and permeability $\left({µ}^{*}\right)$; these dielectric and magnetic properties depend, definitely, on the used absorbing loads in the composite materials. Actually, to optimize the microwave absorption performance of the composite materials, studies focused not only on the composition of the loads, but also on other parameters of these loads. It is shown, for example, that besides the load content [15] and the load shape [16], the load size has a significant influence on the EM absorption performance [16,17,18,19,20].

Studies on the load size have gained much attention with the aim of optimizing the microwave absorption performance. Usually, for these studies, spherical metals or alloy particles are used as absorbing loads [16,17,18,19]. For example, Liu et al. [19] have studied the influence of FeSi particle size on the microwave absorption properties; they showed that both ε′ and ε″ increase with a decrease in particle size. Liu et al. have also shown that the matching frequency f_m_, which describes the position of the absorption peak, shifts towards lower frequency range by decreasing the particle size of FeSi powder. The same results were obtained in the work of Wang et al. using the Fe-Cr-Si-Al alloy flakes [16]. Hong et al. [20] have investigated the influence of particle size, using carbon fibers, on dielectric properties of CFs-loaded epoxy resin composites; their emphasis was on the effect of CF length on the permittivity of the composites. Unlike the results obtained with metallic particles, they measured a higher complex permittivity for the composites loaded with the longest fibers. The relation between CFs length and the complex permittivity has been theoretically demonstrated, based on the Reynolds–Hugh theory [20]. However, the results did not show any relation between dielectric properties and the frequency band, as a function of fiber length in the studied frequency range (8 GHz to 12 GHz).

Furthermore, the dispersion of carbon fillers, such as long CFs, in the matrix is another aspect that needs much attention; numerous works were focused on the improvement of this carbon dispersion [21,22,23]. The mechanical techniques are one of the techniques that are used for the CFs dispersion. For example, in the work of Wang et al. [22], homogenous and un-oriented (3D) dispersion of CFs was achieved in the Si/Phenolic resin by mechanically stirring the solution for 30 min. A good dispersion of CFs within the air-in-resin liquid foam was also achieved in the work of Song et al. [21] after only 2 min of mixing time with a mechanical frothing process. Benzerga et al. [24] have shown the elaboration of homogeneous CFs-loaded composites using the mechanical dispersion method of CFs, but they have also shown CFs breakage with this dispersion technique. Moreover, ultrasounds are also used for the dispersion of CFs [25,26,27]. Chuang et al. [26] have demonstrated that the pre-dispersion of CFs in water by ultrasonic vibration for 15 min greatly improves the final CFs-loaded material. In the work of Nanni et al. [25], carbon nanofibers were pre-dispersed into different solvents under sonication for 1 h, to achieve homogenous carbon nanofibers/epoxy composites. None of both works has mentioned the damage of CFs after ultrasounds dispersion.

The aim of this work is to elaborate broadband absorbing material operating in the frequency range between 1 and 15 GHz. The relation between the CFs length and the microwave absorption performance, especially the absorption frequency band will be studied. For this purpose, the dielectric and microwave absorption properties of epoxy foam composites loaded with low weight percentages (≤0.5 wt.%) and different lengths of CFs, which are obtained with three methods of CFs dispersion, will be presented. In this work, the effect of the breakage of CFs on the absorption performance of the composite will be experimentally demonstrated. Therefore, composites loaded with different CF lengths (3, 6, and 12 mm) were elaborated in order to confirm the relation between CF lengths and dielectric properties. Then, hybrid material, elaborated using a mix of CF lengths, is proposed and its dielectric properties are compared to the previous composites. Finally, simulations of absorption performance of pyramidal absorbers are conducted using the dielectric properties of the different elaborated composites made with CF lengths ranging between 3 mm and 12 mm.

## 2. Materials and Methods

### 2.1. CFs/Epoxy Foam Composite Elaboration

**Figure 1:**
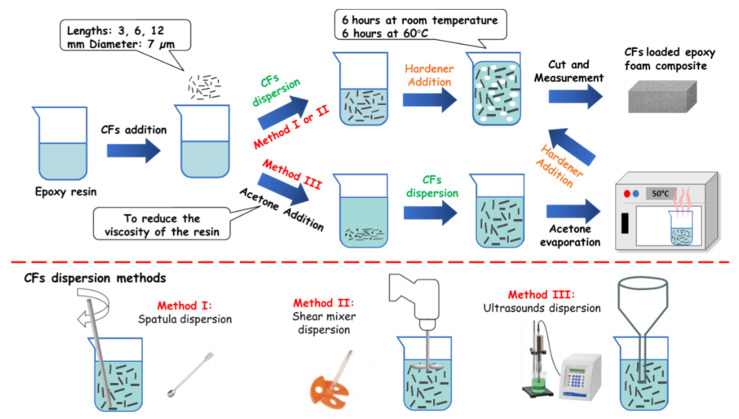
Schematic diagram of the elaboration method of the carbon fibers (CFs)-loaded epoxy foam composites and the used CFs dispersion methods.

The CFs-loaded epoxy foam composites were prepared using PB170 epoxy resin and DM02 hardener (foaming agent) purchased from Sicomin. CFs, of 7 µm diameter and of different lengths (3 mm, 6 mm, and 12 mm) from Apply Carbon (Figure S1), were used as absorbing loads. Figure 1 shows the different steps of the elaboration method of composites. First, the chosen weight percentage of CFs was added to the epoxy resin and dispersed using one of the three used methods: spatula (method I), shear mixer RYOBI EID 100 2RE model (method II), and Ultrasounds VCX-750-220 from Sonics Materials (method III). It should be noted here that for method III, the viscosity of the resin was reduced using acetone (50 %V. of resin and 50 %V. of acetone) before the sonication step and pulses of 300 W of power were applied. Then, the mixture was placed in the oven at 60 °C for 72 h to evaporate the acetone completely before adding the hardener. Finally, whatever the used dispersion method, the hardener was added to the mixture with a 10/3.6 resin/hardener ratio. The final mixture remained at ambient temperature within 6 h for the foaming process and polymerization step and then, it was cured at 60 °C for at least 6 h to achieve the polymerization of the epoxy foam composites. After the curing step, samples were cut to 15 × 15 × 6 cm^3^ for the characterization in the anechoic chamber.

### 2.2. Structural Characterization

The surface morphology of the CFs and the CF-loaded epoxy foam were analyzed by an optical microscope Leica DM 2500 M and a scanning electronic microscope (SEM) JEOL 5600. Composite densities were calculated according to the following formula: $\rho =\frac{m}{V},$ where m is the weight and V is the volume of the samples. A measurement, uncertainty of ±0.01 g·cm^−3^ was estimated and taken into account for the presented density values.

### 2.3. Dielectric and EM Characterization

**Figure 2:**
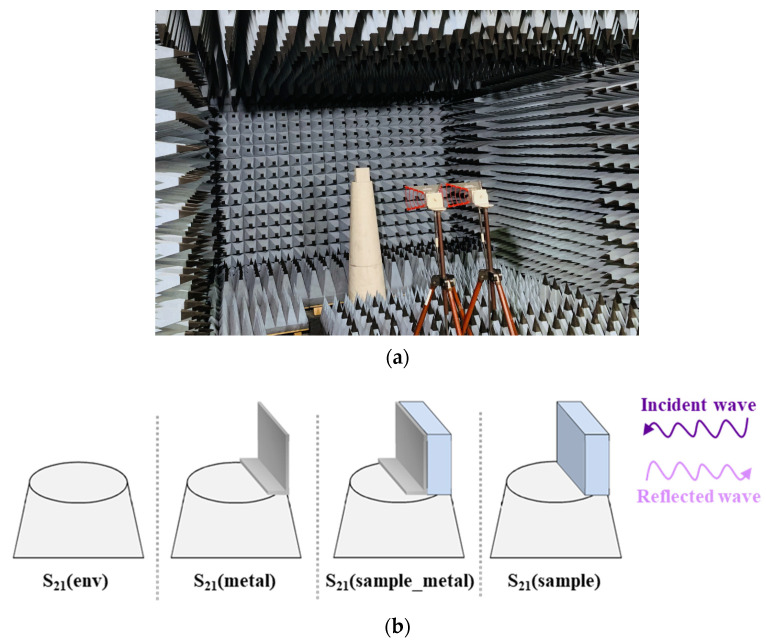
Measurement configurations (**a**) in the anechoic chamber and (**b**) of the four different S_21_ parameters.

The dielectric and EM characterization of the composites were carried out using the free space method [28]. A sample with dimensions of 15 × 15 × 6 cm^3^ was placed inside the anechoic chamber (Figure 2a) in front of two horn antennas (3115 Ets-Lindgren Model) connected to a vector network analyzer (VNA). The sample was placed at a distance d from the antennas, ensuring the far field condition, (Equation (1)) [29]:

(1) $$d\geq \frac{2D^{2}}{\lambda },$$

where D is the antenna aperture diameter and λ is the highest measured wavelength.

This configuration was used for the measurement of the reflection coefficient and for the extraction of the dielectric properties in the frequency range of 1–15 GHz.

#### 2.3.1. The Reflection Coefficient Measurement

Four different S_21_ parameters were measured in the anechoic chamber: the S_21_ of the sample alone ($S_{21}\left(\mathrm{sample}\right))$, the S_21_ of the sample with metallic plate $\left(S_{21}\left(\mathrm{sample}\_\mathrm{metal}\right)\right)$, the S_21_ of the metallic plate alone $\left(S_{21}\left(\mathrm{metal}\right)\right),$ and the S_21_ of the environment $\left(S_{21}\left(\mathrm{env}\right)\right)$ as shown in Figure 2b. The reflection coefficients Γ of the material alone (Γ_sample_) and of the material with a metallic plate (Γ_sample_metal_) are calculated, using the four measured S_21_, as follows:

(2) $$\Gamma_{\mathrm{sample}}=\frac{S_{21}\left(\mathrm{sample}\right)-S_{21}\left(\mathrm{env}\right)}{S_{21}\left(\mathrm{env}\right)-S_{21}\left(\mathrm{metal}\right)},$$

(3) $$\Gamma_{\mathrm{sample}\_\mathrm{metal}}=\frac{S_{21}\left(\mathrm{sample}\_\mathrm{metal}\right)-S_{21}\left(\mathrm{env}\right)}{S_{21}\left(\mathrm{env}\right)-S_{21}\left(\mathrm{metal}\right)}.$$

It should be noted here that all the installed absorbers in the anechoic chamber (targeted application) are backed by a metallic plate. Therefore, the reflection coefficients presented in the next paragraphs are the reflection coefficients of the materials backed with a metallic plate Γ_sample_metal_. In order to simplify the reading of this article, the Γ_sample_metal_ parameter will be named Γ.

#### 2.3.2. Extraction of Dielectric Properties

The complex permittivity $\left(\varepsilon_{r}^{*}=\varepsilon_{r}^{\prime }-{j\varepsilon }_{r}^{\prime \prime }\right)$ of the characterized materials is extracted from the measured reflection coefficients using Fenner et al.’s method [30]. The impedance of the material alone, $Z^{*}{}_{\mathrm{sample}},$ and the impedance of the material with the metallic plate, $Z^{*}{}_{{\mathrm{sample}}_{\mathrm{metal}}},$ are calculated as follows:

(4) $$Z_{\mathrm{sample}}^{*}=Z_{0}*\left(\frac{1+\Gamma_{\mathrm{sample}}}{1-\Gamma_{\mathrm{sample}}}\right),$$

(5) $$Z_{\mathrm{sample}\_\mathrm{metal}}^{*}=Z_{0}*\left(\frac{1+\Gamma_{\mathrm{sample}\_\mathrm{metal}}}{1-\Gamma_{\mathrm{sample}\_\mathrm{metal}}}\right),$$

where $Z_{0}=377\;\Omega$ is the characteristic impedance of air.

As our composites are dielectric materials, the magnetic properties are not considered. The complex permittivity is then calculated as follows:

(6) $$\varepsilon_{r}^{*}=\frac{Z_{0}^{2}}{{\tilde{Z}}^{2}}+\sin^{2}\theta ,$$

where θ is the angle of incidence of EM wave used for the measurement and

(7) $$Z^{*2}=\frac{Z_{0}{*Z}_{\mathrm{sample}\_\mathrm{metal}}{*Z}_{\mathrm{sample}}}{Z_{0}+Z_{\mathrm{sample}\_\mathrm{metal}}-Z_{\mathrm{sample}}}.$$

Dielectric losses are defined by $\tan\delta =\varepsilon_{r}^{\prime \prime }/\varepsilon_{r}^{\prime }$. They indicate the overall microwave attenuation inside the composite material.

#### 2.3.3. Simulation of Absorption Performance of Pyramidal Absorber

The absorption performance of composites, in the frequency range 1–15 GHz, is estimated by the simulation of the reflection coefficients of pyramidal absorbers. These simulations were carried out using the CST Microwave Studio software and the measured dielectric properties of the composites. The typical formulas used for the calculation of the reflection coefficient of planar absorber backed with a metallic plate are as follows:

(8) $$\Gamma \;\left(\mathrm{dB}\right)=20\log|\frac{Z_{\mathrm{in}}-1}{Z_{\mathrm{in}}+1}|,$$

(9) $$Z_{\mathrm{in}}\left(\Omega \right)=Z_{0}\sqrt{\frac{\mu_{r}^{*}}{\varepsilon_{r}^{*}}}\tanh\left(j\left(\frac{2\pi \mathrm{fd}}{c}\right)\sqrt{\varepsilon_{r}^{*}\mu_{r}^{*}}\right),$$

where Z_in_ is the normalized input impedance and Z_0_ is the characteristic impedance of the vacuum (377 Ω). $\mu_{r}^{*}$ is the relative complex permeability ($\mu_{r}^{*}=\frac{\mu^{*}}{\mu_{0}}={\text{µ}}_{r}^{\prime }-{j\text{µ}}_{r}^{\prime \prime })$ of the material (equal to 1 for our dielectric materials), $\varepsilon_{r}^{*}$ is the relative complex permittivity of the material ($\varepsilon_{r}^{*}=\frac{\varepsilon^{*}}{\varepsilon_{0}}=\varepsilon_{r}^{\prime }-{j\varepsilon }_{r}^{\prime \prime })$, with $\varepsilon_{0}$ and $\mu_{0}$, the permittivity and permeability of air, respectively. f is the frequency of the incident wave, d is the thickness of the material, and c is the speed of light in vacuum.

In this work, the pyramidal geometry APM12 of SIEPEL [3] is used for simulations. The dielectric properties, the shape, and dimensions of the pyramid are introduced in the CST software, and the Floquet boundary conditions are applied in order to simulate an infinite number of pyramids. In the case of pyramidal absorber, formulas shown in [31] are then taken into account by the software.

## 3. Results and Discussions

### 3.1. Effect of CFs Breakage on Reflection Coefficient of CFs Loaded Composites

#### 3.1.1. Effect of CFs Dispersing Method

**Figure 3:**
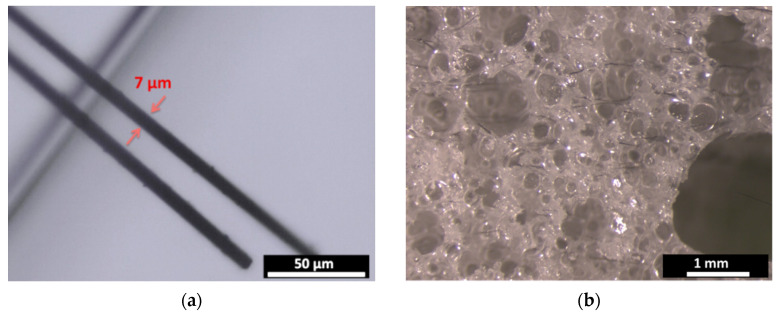

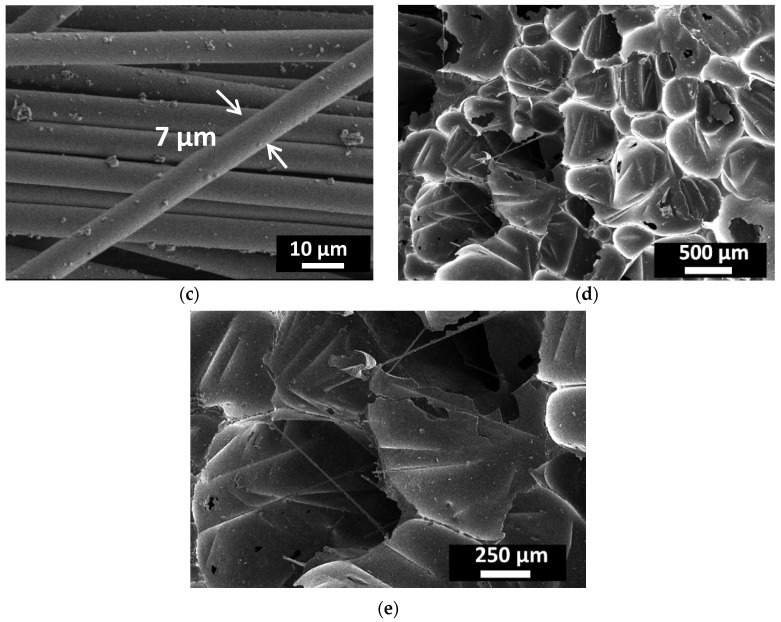
Optical micrographs of (**a**) 3 mm-CFs and (**b**) CFs-loaded epoxy foam, and SEM images of (**c**) 3 mm-CFs, (**d**) and (**e**) CFs-loaded epoxy foam. The presented composite is loaded with 0.5 wt.% of 3 mm-CFs dispersed with spatula.

Three samples of epoxy foam loaded with 0.5 wt.% of CFs of 3 mm length are prepared using the different CFs dispersing methods for 5 min. Figure 3a,b shows the optical micrographs of the used 3mm-CFs (Figure 3a) and CFs-loaded epoxy foam prepared using the spatula method (Figure 3b). SEM images of these CFs and the same elaborated composite are presented in Figure 3c,d, respectively. The CFs-loaded epoxy foam shows a wide and a random porosity distribution ranging from few tens of micrometers to few millimeters. The carbon fibers are also randomly distributed in the epoxy foam and can show orientations in the three directions of space (Figure 3e). It should be noted here that these macroscopic materials seem the same and present the same microstructure when they are observed by SEM or by optical microscope. For this reason, photos showing the whole samples were preferred for this paper.

**Figure 4:**
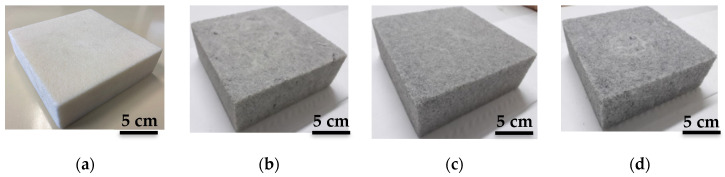
Photos of (**a**) unloaded epoxy foam and samples loaded with 0.5 wt.% of 3 mm-CFs made with (**b**) spatula dispersion, (**c**) shear mixing, and (**d**) ultrasounds. Mixing duration is 5 min.

Figure 4 presents photos of the unloaded epoxy foam (Figure 4a) and 3 mm-CFs-loaded epoxy foam composites, elaborated using different CF dispersing methods: spatula (Figure 4b), shear mixing (Figure 4c), and ultrasounds (Figure 4d). Unlike the unloaded foam, the loaded samples show a gray coloration provided by the addition of CFs. It should be noted here that a homogeneous gray color reflects a good homogeneity (good CF dispersion) of the elaborated sample. Taking into account the initial agglomerated appearance of the used fibers shown in Figure S1, the elaborated samples exhibit a good homogeneity; these samples present an identical measured density equal to ρ=0.13 g·cm^−3^.

**Figure 5:**
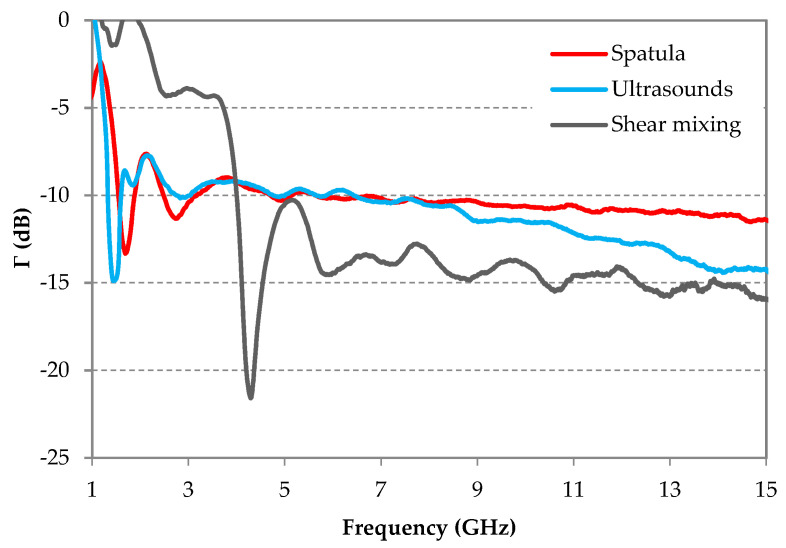
The frequency dependence of the measured reflection coefficients of epoxy foams loaded with 0.5 wt.% of 3 mm-CFs and prepared with the three methods of process: spatula, ultrasounds, and shear mixing.

The elaborated CF-loaded composites, made with three CF dispersion methods, were characterized in the anechoic chamber. Figure 5 shows the reflection coefficients Γ of these samples in the frequency range between 1 and 15 GHz. This figure shows that ultrasounds and spatula fiber dispersion methods provide better absorption performances (lower reflection coefficient) at low frequencies (below 4 GHz), compared to the sample elaborated with the shear mixing method. In fact, in this frequency range, the minimum values of the measured reflection coefficients are Γ = −14.5 dB (at 1.4 GHz) and Γ = −13.9 dB (at 1.55 GHz) for samples elaborated using ultrasounds and spatula methods, respectively. For the shear mixing method, a reflection coefficient around Γ ≈ 0 dB is obtained at the same frequencies; however, for higher frequencies (from 6 GHz), this method shows the best (lowest) reflection coefficient, i.e., Γ < −14 dB between 10 and 15 GHz. For this frequency range, the sample made with shear mixing presents the lowest values of the reflection coefficient, followed by the sample made with ultrasounds, while the sample made with the spatula shows the highest reflection coefficient. For example, at 14 GHz, the measured reflection coefficients are −11 dB, −14.7 dB, and −15 dB for samples made with spatula, ultrasounds, and shear mixing, respectively. To summarize, the samples elaborated using spatula or ultrasound methods present the best reflection coefficient results at low frequencies, while that elaborated using the shear mixing shows the best results at high frequencies.

Furthermore, it has been shown that the use of mechanical (shear) methods for fiber dispersion induced fiber damage [32,33]. For example, in [32], after 2 min of mixing time, the average length of fibers decreased from 3.5 mm to around 0.7 mm with 80 rpm of rotor speed. Likewise, the use of ultrasounds reduces, but with a smaller extent, the length of the fibers [24]. In order to confirm the effect of CFs mixing methods on the fiber length, photos of CFs after the mixing step were done.

**Figure 6:**
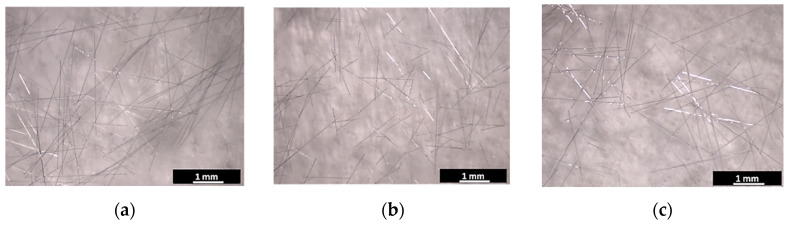
Microscopic images of 3 mm-CFs mixed with (**a**) spatula, (**b**) shear mixing, and (**c**) ultrasounds for 5 min.

Figure 6 shows the microscopic images of 3 mm-CFs dispersed during 5 min with one of the three different used methods (spatula, shear mixing, and ultrasounds). In our case, the length of the fibers was not damaged at all when they are dispersed with a spatula (Figure 6a), while it was largely damaged using shear mixing, as shown in Figure 6b; CFs dispersed using ultrasounds appear less damaged (Figure 6c). Indeed, most of the CFs present a length lower than 1 mm in Figure 6b while only few CFs have this length in Figure 6c. These observations suggest that the length of CFs is finally much smaller in the sample elaborated with the shear mixing method than in the one elaborated with ultrasound, as well as the sample elaborated with the spatula method. According to these results, the observed reflection coefficient behavior of samples made with different dispersion methods, as a function of frequency (Figure 5), may be related to the length of fibers in the obtained composites.

#### 3.1.2. Effect of CFs Shear Mixing Duration

**Figure 7:**
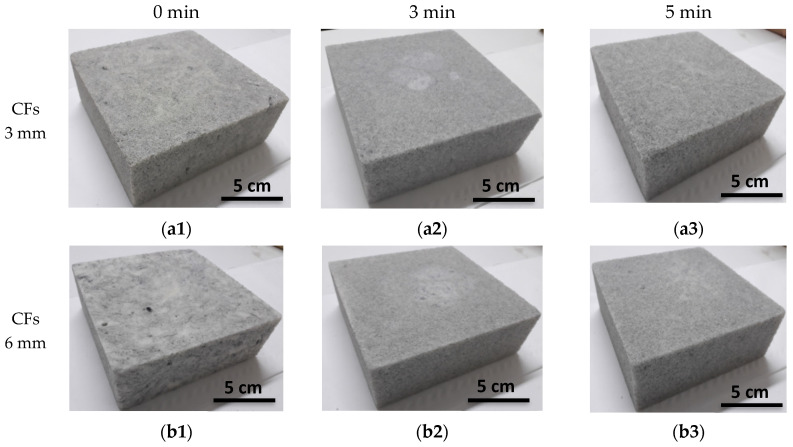
Epoxy foam loaded with 0.5 wt.% of (**a**) 3 mm-CFs and (**b**) 6 mm-CFs prepared with shear mixing for (**1**) 0 min, (**2**) 3 min, and (**3**) 5 min.

In order to confirm the relationship between absorption performance and fiber breakage, and therefore fiber length, samples made with the shear mixing method (Figure 7) were prepared using different dispersion times (0 min, 3 min, and 5 min). These different dispersion times were applied in order to obtain different CF lengths; here, 0 min corresponds to 5 min of mixing with spatula. Three initial fiber lengths (3 mm, 6 mm, and 12 mm) were used for this part (Figure S1).

Figure 7 shows the different samples loaded with 0.5 wt.% of 3 mm-CFs (Figure 7a) and 6 mm-CFs (Figure 7b) and with different mixing times (0 min, 3 min, and 5 min). Samples that are prepared with spatula (0 min) show some CF aggregates (Figure 7(a1,b1)), identifiable by a locally darker gray color, as shown in Figure 7(b1) (sample made with 6 mm-CFs). The other samples present a homogeneous appearance, especially those obtained by 5 min mixing time (Figure 7(a3,b3)). A better homogeneity, thus a better dispersion of CFs, is therefore obtained with shear mixing, particularly, with the highest mixing time (5 min), probably due to the CF breakage.

Figure S2 shows the microscopic images of CFs (3 mm, 6 mm, and 12 mm) after 0 and 5 min of mixing time. This figure shows that most CFs resulting from the spatula mixing (0 min) preserve their initial lengths of 3 mm (Figure S2(a1)), 6 mm (Figure S2(a2)), and 12 mm (Figure S2(a3)). After 5 min of shear mixing, the length of 3 mm-CFs appears to be between 0.5 and 3 mm with an estimated average CF length around 1 mm (Figure S2(b1)). Furthermore, Figure S2(b2,b3) show clearly that whatever the initial length of CFs is, the final length of these fibers, after 5 min of shear mixing, is around 1 mm. Therefore, the CFs are totally damaged after 5 min of shear mixing and present, at the end, approximately the same lengths except the few unbroken fibers that can probably subsist.

It should be noted here that the breakage of CFs is random; hence, the length of different fibers in samples is not quantifiable. Therefore, we assume that the longer the mixing time, the higher the damage of the fiber length, and consequently, the shorter the fiber length.

**Figure 8:**
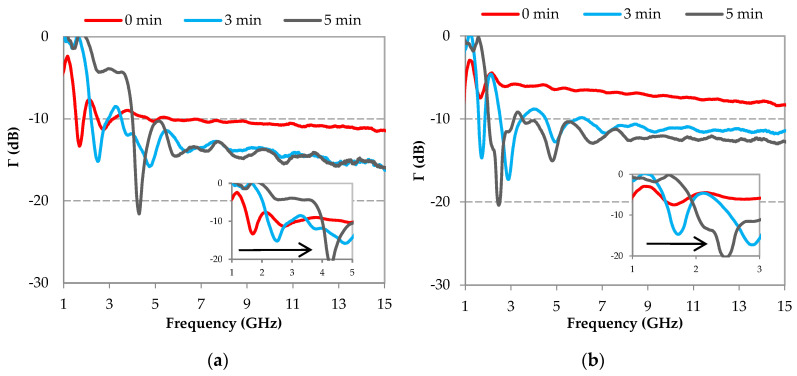
Frequency dependence of the measured reflection coefficient for epoxy foams loaded with 0.5 wt.% of (**a**) 3 mm-CFs and (**b**) 6 mm-CFs obtained with three times of shear mixing: 0 min, 3 min, and 5 min.

Figure 8 shows the measured reflection coefficients of the elaborated samples, as a function of the frequency. This figure highlights, as before (Figure 5), two different behaviors according to the considered frequency range; in other words, samples with the lowest reflection coefficients at low frequencies present the highest coefficients at high frequencies, and vice versa. For example, samples loaded with 3 mm-CFs (Figure 8a) show the lowest reflection coefficient for the sample made with 0 min shear mixing time at low frequencies (Γ = −12.23 dB @ 1.6 GHz), while the same sample presents the highest reflection coefficient at high frequencies (−11.4 dB < Γ < −10 dB for frequencies f > 6 GHz). Unlike that, for the same CF length (3 mm), the sample made with 5 min of shear mixing presents the highest reflection coefficient at low frequencies (−5 dB < Γ < 0 dB for frequencies f < 4.4 GHz) and lowest reflection coefficient at high frequencies (−16 dB < Γ < −14.5 dB for frequencies f > 6 GHz). It is interesting that, with the extension of mixing time from 0 to 5 min, the peak of the reflection coefficient is shifted to high frequencies (as schematized by the black arrow); this shift is more clearly observed in the zoom inserted in Figure 8a. This shift is comparable to that observed previously in Figure 5, when different mixing techniques were used. The same observation can be made on samples loaded with 6 mm-CFs (Figure 8b). Moreover, beyond 6 GHz, the reflection coefficients show a decrease of approximately 5 dB, before (0 min) and after 5 min of shear mixing; this result was obtained whatever the initial length of CFs (Figure 8).

To summarize, results have shown that when the shear mixing time is extended to 5 min, the length of CFs decreases, the peak of the reflection coefficient shifts toward the high frequencies, and the reflection coefficient, at these high frequencies, decreases. This observation is consistent with the previous results (Figure 5) and further highlights a relationship between the CF breakage (CF length) and the microwave properties (here, reflection coefficient).

It should be noted here that the level of the reflection coefficient is globally lower for samples loaded with 3 mm-CFs compared to samples loaded with 6 mm-CFs. For example, at 10 GHz, Γ = –10.60 dB and Γ = −7.28 dB are obtained for samples mixed with spatula (0 min) and loaded with 3 mm-CFs and 6 mm-CFs, respectively (Figure 8). This higher reflection coefficient for 6 mm-CFs composites is probably due to their higher conductivity induced by a lower percolation threshold for these composites. Indeed, the electrical conductivity of carbon-loaded composites depends not only on the rate, but also on the dimensions of the carbonaceous load [34,35,36]. The longer the CF length, the easier the CF fillers exceed the percolation threshold, in other words, a conductive network is easily formed inside the composite [37,38]. It should be noted here that the high conductivity of the composite increases the reflection of the incident wave at the surface of the material due to an impedance mismatch at the air/absorber interface. The increase of the wave reflection at the interface results in a decrease in the absorption performance of the absorbing material. An impedance matching can be ensured by a compromise between the length and the content of CF loads.

Figures S3 and S4 present the photos of the elaborated composites loaded with 12 mm-CFs, using the shear mixing method for different times (Figure S3), and the measured reflection coefficients of these samples (Figure S4). Note that in the case of 12 mm-CFs, the 0 min mixing time was replaced by 30 sec because of the agglomerated aspect of these fibers (CF bundles shown in Figure S1), which makes it impossible to disperse them by spatula. This can be observed on the sample elaborated with spatula (Figure S3a); it has the same white color as an unloaded sample (Figure 4a). Moreover, with these long CFs, the elaborated samples present a highly inhomogeneous appearance, especially for samples made with a mixing time equal to or less than 3 min (Figure S3), unlike the elaborated samples with shorter fibers (3 and 6 mm). However, the measured reflection coefficients of these samples (Figure S4) present exactly the same behavior observed before, that is, a shift of the minimum reflection coefficient peak towards the high frequencies when the mixing time increases, with an improvement of the reflection coefficient at high frequencies.

Our results show that the increase of the shear mixing time is accompanied by a deterioration of CFs; hence, a decrease in their lengths. We can hypothesize that the observations made on the reflection coefficients, as a function of the mixing time, are related to the effect of fiber lengths. Since the reflection coefficient of an absorber is related to its dielectric properties, the length of the fibers has surely an effect on these dielectric properties.

### 3.2. Effect of Carbon Fiber Lengths on Dielectric Properties of CF-Loaded Composites

To study the effect of CF length on the dielectric properties of our materials, three samples loaded with three different CF lengths (3 mm, 6 mm, and 12 mm) are prepared and measured in free space. The dielectric properties of these samples were extracted using the method detailed in paragraph (2.3.2). It should be noted here that for these composites, CFs are dispersed using ultrasounds. This method provides the best compromise between the homogeneity of the composites, as shown in the photos of the elaborated samples (Figure S5), and the low damage of the CF length, as confirmed by the optical micrographs of the used fibers (3, 6, and 12 mm-CFs) after ultrasound dispersion for 5 min (shown in Figure S6). These optical micrographs confirm that CF length is generally preserved whatever the initial CF length. Otherwise, epoxy foams loaded with only 0.25 wt.% were chosen for this study to avoid a conductive behavior of the samples. In fact, our previous work has shown that composites loaded with 12 mm-CFs show a very low percolation threshold (below 0.5%) and become conductive [39].

**Figure 9:**
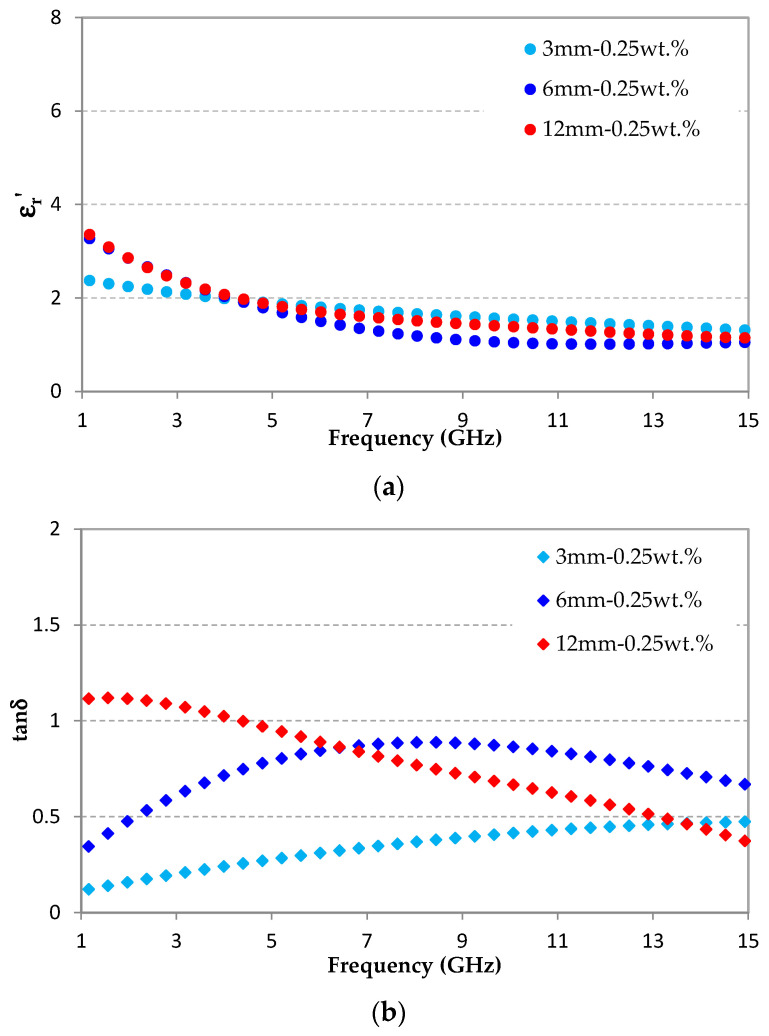
Dielectric properties of epoxy foams loaded with 0.25 wt.% of 3, 6, and 12 mm-CFs: (**a**) real part of the relative permittivity and (**b**) dielectric losses.

Figure 9 shows the dielectric properties—real part of the relative permittivity (Figure 9a) and dielectric losses (Figure 9b)—extracted from the measurement in anechoic chamber of samples loaded with 0.25 wt.% of 3, 6, and 12 mm-CFs. Figure 9a shows that the real parts of the permittivities are very close and do not show any logical reasoning between the three samples as a function of the CF length. Therefore, here, the real permittivity seems weakly affected by the CF length. However, a study of Hong et al. has shown a slight effect of CF length on the real part of the permittivity of composites based on epoxy resin loaded with CFs of different lengths [20]. This study deals with a resin, which ensures an alignment of CFs (or, at least, a privileged orientation of the latter), unlike the random orientation of CFs imposed by the foam matrix in our foam-based composites (Figure 3d). Otherwise, for our materials, the low rate of CFs (≤0.5 wt.%) and the contribution of air (provided by the porosity of the foam) induce a low real permittivity. Indeed, beyond 6 GHz, the samples show low permittivity values ($\varepsilon_{r}^{\prime }$ ≤ 1.7) close to the air permittivity ($\varepsilon_{0}$ = 1).

From the extracted dielectric losses of our materials, shown in Figure 9b, it is clearly visible that the maximum of the dielectric losses is obtained at low frequencies for material loaded with 12 mm-CFs (tanδ_max_ = 1.12 @ 2 GHz) and at high frequencies for material loaded with 3 mm-CFs (tanδ_max_ = 0.47 @ 15 GHz). Additionally, the material loaded with 6 mm-CFs shows maximum losses at the central frequencies, tanδ_max_ = 0.89 @ 8 GHz. Consequently, this result confirms the effect of the CF length on the absorption frequency range, represented here by the dielectric losses. Therefore, long CFs are more interesting for absorption at low frequencies while short CFs (3 mm) are more interesting for absorption at high frequencies. This result is consistent with the previous observations (Figure 5 and Figure 8). Indeed, the reflection coefficients are lower at low frequencies for samples containing long fibers (samples prepared with spatula); and at high frequencies, the best coefficients are obtained for samples containing short fibers (fibers dispersed by shear mixing during 5 min).

To the best of our knowledge, different studies have shown the effect of fiber length on dielectric losses [20,40], but there are no studies to date that have shown a relationship between the fiber length and the frequency, where the maximum losses occur.

For our composite materials based on polymer foam loaded with carbon fibers, we assume that dielectric losses result from several phenomena: polarization, conduction, as well as multi-reflection and backscattering phenomena. The dielectric losses induced by polarization in CF-loaded composites increase when CF length increases [20]. The conduction losses also increase when the CF length increases [37]; this is often associated to the decrease of the percolation threshold with the increase of the CF length [20,37]. One can note that a 3D distribution of CFs inside the matrix also contributes to the decrease of the percolation threshold [41]. On the other hand, the presence of eddy currents, in carbon fibers exposed to the alternating magnetic fields of the EM waves, could induce significant dielectric losses [42,43]. These losses increase when the rate of carbon fibers increases due to a coupling effect between the fibers [42]. However, the cited studies do not observe any variation of the induced dielectric losses in function of fiber lengths versus frequency, but it should be noted here that for all the cited references [20,43], short fibers (length between 1 to 3 mm) were used and studied in the X-band frequency range. A zoom of our dielectric properties in this same frequency range, presented in Figure S7, shows that the dielectric losses seem practically constant and vary very slightly in this range; the 6 mm-CFs show the highest values because, precisely, their maximum losses are in this frequency range (as shown in Figure 9).

Otherwise, multi-reflections and backscattering occur in a porous matrix, between the air/matrix [9,44]; these losses are negligible compared to the measured losses of our composites. Multi-reflections occur also between the matrix and the fibers [35] and the attenuation of the waves (by trapping) in fiber agglomerates is expected [45]. In our composites, the shape and the dimensions of the agglomerates are random, but we can suggest that the crossed long CFs, will trap and absorb (by multi-reflections) the EM waves of long wavelengths (low frequencies) as schematized in Figure S8a; while small EM wavelengths (at high frequencies) will be able to pass through these agglomerates (Figure S8b). Contrary to this, the agglomerates, which are formed by small fibers, will be able to trap small wavelengths (Figure S8c), but not long wavelengths that can be either reflected by these agglomerates (Figure S8d) or simply pass without being affected by them (blue arrows in Figure S8). For this reason, short and long fibers probably show a maximum of their dielectric losses at high and low frequencies, respectively.

### 3.3. Towards a Hybrid Absorber Composite Material

To achieve an absorbing material that performs over a wide frequency range and is also effective at low frequencies, hybrid material based on the combination of different CF lengths was elaborated and measured in the frequency band of 1–15 GHz. The longest CFs have shown the best dielectric properties at low and medium frequency ranges before. Therefore, in this section, 12 mm-CFs and 6 mm-CFs are chosen as loads. Thus, a sample loaded with a combination of long CFs (6 mm + 12 mm) and a load rate of 0.25 wt.% (0.125 wt.% of each type of CFs) was prepared (Figure S9); CFs were dispersed using the ultrasounds method.

**Figure 10:**
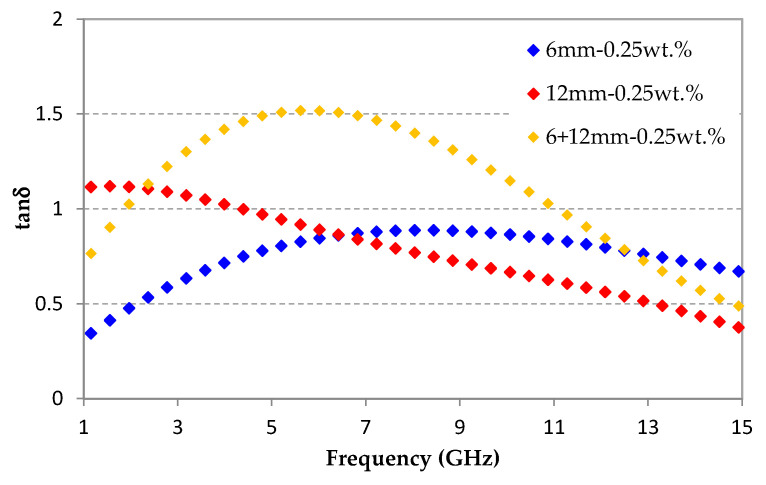
Dielectric losses of epoxy foams loaded with 0.25 wt.% of 6, 12, and 6 + 12 mm-CFs.

Figure 10 shows the dielectric losses of this hybrid sample as a function of frequency. These dielectric losses are compared with the losses of the two samples loaded with 6 mm-CFs and 12 mm-CFs. This figure shows that the maximum of dielectric losses of the hybrid material is around the frequency of 5.6 GHz—it is between the two maximums of dielectric losses obtained for samples loaded with 6 mm-CFs and 12 mm-CFs at 2 and 8 GHz, respectively. The highest dielectric losses at low frequencies are obtained for materials filled with 12 mm-CFs, which contains 0.25 wt.% of these fibers, compared to the hybrid material that contains, at the end, only 0.125 wt.% of these long fibers. Moreover, and as expected, the combination of different CF lengths gives interesting dielectric losses over a large frequency band, ranging between tanδ = 0.77 (@ 1 GHz) and tanδ = 0.49 (@ 15 GHz), with a maximum value of tanδ_max_ = 1.51 (@ 5.6 GHz). These high dielectric losses are very interesting with regard to the targeted application of absorbing material.

### 3.4. Simultaion of Absorption Performance of Pyramidal Absorber Based on Hybrid Composite

**Figure 11:**
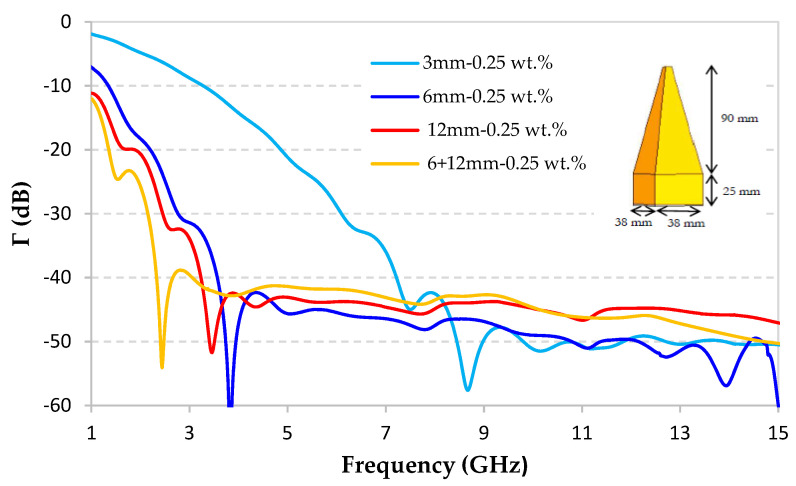
Simulated reflection coefficient of pyramidal absorbers performed using measured dielectric properties of epoxy foams loaded with 0.25 wt.% of 3, 6, 12 and 6 + 12 mm of CFs.

In this section, the absorption performance of pyramidal absorber based on hybrid material is estimated for the application in anechoic chambers. For this reason, the geometry of the commercial pyramidal absorber APM12 from Siepel [3] is used (Figure 11). It consists of pyramids with a square base of 38 × 38 mm^2^ and a height of 90 mm resting on a 25 mm thick base. Therefore, the total height of the pyramid is 115 mm. This geometry was chosen because it is used in anechoic chambers for the frequency band 2–20 GHz [3]. The aim of this work is to use the same geometry (and height) of the pyramidal absorber and to ensure a good absorption performance for frequencies, lower than 2 GHz. Otherwise, as mentioned before, to ensure a minimum reflection, so a maximum absorption, of the incident EM waves, an impedance matching between the air and the absorber is firstly needed. In the case of pyramidal absorber and contrary to the planar absorbers, this impedance matching is ensured by the gradual (pyramidal) shape of the material [46]. Therefore, for this design, high dielectric losses are required to ensure the absorption at low frequencies, without increasing the dimensions of the pyramids. Note that for absorbing materials, a reflection coefficient lower than −10 dB is needed, which indicates that the absorption of the incident EM waves is greater than 90%.

Figure 11 shows the simulated reflection coefficient of pyramidal absorbers obtained using measured dielectric properties of composites loaded with 0.25 wt.% of 3, 6, 12, and 6 + 12 mm-CFs. Results show that a composite loaded with 12 mm-CFs provides a very good reflection coefficient at low frequencies. Indeed, at 1 GHz, a Γ = −12 dB and −11 dB is obtained for composites loaded with 6 + 12 mm-CFs and 12 mm-CFs, respectively. Whereas, at 1 GHz when the composite is loaded with only 3 mm-CFs or 6 mm-CFs, this reflection coefficient Γ increases to −7 dB and −3.7 dB, respectively. At the high frequencies, beyond 7 GHz, all the absorbing materials show a reflection coefficient below −40 dB. As explained above, this type of absorbers shows a high performance at high frequencies owing to the height and the pyramidal geometry of this absorber. However, for intermediate frequencies (between 4 GHz and 7 GHz), a composite loaded with only 3 mm-CFs shows the highest reflection coefficient, and thus lowest absorption, induced by its low dielectric losses (tanδ < 0.35). For this composite, a higher pyramidal absorber is required in order to enhance its absorption at the low frequency band. Finally, Figure 11 confirms that for the chosen pyramidal geometry, a better absorption performance can be obtained, especially in the low frequency band, for the hybrid material.

For this study, we were not able to achieve a pyramidal absorber prototype because this one is restricted by the requirement of the production and the machining of several tens of pyramids. Nevertheless, we compared our results, in supplementary data (Figure S10), with the performance (measurement and simulation) of the pyramidal absorber prototype of our previous work [14], based on epoxy foam loaded with 0.5 wt.% of 3 mm-CFs. Figure S10 shows, on the one hand, a very good agreement between the simulation (purple dashed line) and the measurement (purple continuous line) results of this previous pyramidal absorber prototype. This confirms that the used simulation method can perfectly predict the absorption performance of pyramidal absorber prototype based on our composite materials. On the other hand, Figure S10 also shows a better predicted performance for our hybrid absorber (0.25 wt.% of 6 + 12 mm-CFs) compared to the other composites, especially at the low frequency band (between 1 and 5 GHz).

Furthermore, and as expected, our previous composite loaded with 0.5% of 3 mm-CFs shows a better performance than that elaborated here with the same fiber length but with a lower percentage of 0.25 wt.%. However, one can note that all composites loaded with 0.25% of long fibers (yellow, red, and dark blue curves) predict a higher absorption performance than the absorber made with a higher CFs rate (0.5 wt.%) of short fibers (3 mm). This confirms the certain interest of long CFs to optimize the absorption performance at low frequencies, and this for very low CF loading rates.

## 4. Conclusions

This work presents CF-loaded composites as microwave absorbers for anechoic chamber application. The relation between the CF length and the microwave absorption performance, especially absorption frequency band, is evidenced. Epoxy foams loaded with different CF lengths (3, 6, and 12 mm) were elaborated using different CF dispersion methods (spatula, shear mixing, and ultrasounds); other composites were prepared using different shear mixing durations (0, 3, and 6 min). Measured reflection coefficients, in free space, of the elaborated samples show that fiber breakage decreases the absorption performance at low frequencies (below 4 GHz), while it improves the absorption at high frequencies. This reveals an influence of the CF length on the absorption frequency band. This influence was confirmed by the measurement of the dielectric properties of composites loaded with different CF lengths (3, 6, and 12 mm). A composite loaded with long CFs shows a maximum dielectric losses at low frequencies (tanδ_max_ = 1.12 @ 2 GHz for 12 mm-CFs), while the composites loaded with short CFs present maximum losses at high frequencies (tanδ_max_ = 0.89 @ 8 GHz and tanδ_max_ = 0.47 @ 15 GHz for 6 mm and 3 mm CFs lengths, respectively).

Furthermore, to obtain a high absorption performance over the whole bandwidth, including low frequencies (lower than 3 GHz), hybrid material loaded with 0.25 wt.% of a combination of two CF lengths (6 + 12 mm) was prepared and characterized. This hybrid material presents high dielectric losses in the entire studied frequency range (0.49 < tanδ < 1.51). The simulated absorption performance of a pyramidal absorber with this hybrid composite is shown and compared to that of the pyramidal absorber simulated using composites loaded with 3, 6, and 12 mm-CFs. Results predict the best performance for the hybrid composite, with a reflection coefficient less than −12 dB in the entire studied frequency band 1–15 GHz, and a reflection coefficient less than −40 dB beyond 3 GHz.

This study confirms that the combination of epoxy foam and millimetric CFs is an extremely promising alternative material for the commercially available absorbers used in anechoic chambers. The use of long CFs brings a very interesting absorption performance, especially at low frequencies, with very low rate loads. The mix of different CF lengths allows an absorption in a large frequency band; thus, ensuring the compactness of the absorbers in anechoic chambers, while maintaining a very good absorption performance.

## Supplementary Materials

The following are available online at https://www.mdpi.com/2072-666X/11/12/1081/s1, Figure S1: Bundles of carbon fibers: (a) 3 mm, (b) 6 mm and (c) 12 mm supplied by Apply Carbon; Figure S2: Microscopic images of (1) 3 mm-CFs (2) 6 mm-CFs (3) 12 mm-CFs mixed with shear mixing during (a) 0 min (spatula) and (b) 5 min; Figure S3: Epoxy foam loaded with 0.5 wt.% of 3 mm-CFs and 6 mm-CFs prepared with shear mixing for (**a**) 0 min, (**b**) 30 s (**c**) 3 min and (**d**) 5 min; Figure S4: Frequency dependence of the reflection coefficient for epoxy foams loaded with 0.5 wt.% of 12 mm-CFs for three times of shear mixing: 30 s, 3 min and 5 min; Figure S5: Photos of epoxy foams loaded with 0.25 wt.% of carbon fiber with length of (a) 3 mm (b) 6 mm and (c) 12 mm, elaborated using ultrasounds, during 5 min, for CFs dispersion; Figure S6: Microscopic images of (a) 3 mm-CFs, (b) 6 mm-CFs and (c) 12 mm-CFs mixed with ultrasounds during 5 min; Figure S7: Dielectric losses of epoxy foams loaded with 0.25 wt.% of 3, 6 and 12 mm-CFs, presented in X-Band frequency range; Figure S8: Proposed multiple reflections and backscattering in CF agglomerates as a function of the CF length and EM wavelength. Trapped waves (Red arrows); transmitted waves (blue arrows); reflected waves (green arrows); Figure S9: Photo of epoxy foams loaded with 0.25 wt.% of 6 + 12mm-CFs; Figure S10: The frequency dependence of the simulated reflection coefficients for pyramidal epoxy foams loaded with 0.25 wt.% of 3, 6, 12 and 6 + 12 mm-CFs compared to the measurement and the simulation of a prototype loaded with 0.5 wt.% of 3 mm-CFs.
