# Supplementary material for: Long Carbon Fibers for Microwave Absorption: Effect of Fiber Length on Absorption Frequency Band
Source: Micromachines (Basel). 2020 Dec 6;11(12):1081. doi: 10.3390/mi11121081 (PMC7762112; doi:10.3390/mi11121081)
Supplement: Supplementary file 1 [file micromachines-11-01081-s001.pdf]

micromachines

Supplementary Materials: Long Carbon Fibers for Microwave Absorption: Effect of Fiber Length on Absorption Frequency Band

Hanadi Breiss, Aicha El Assal, Ratiba Benzerga *, Chloé Méjean and Ala Sharaiha

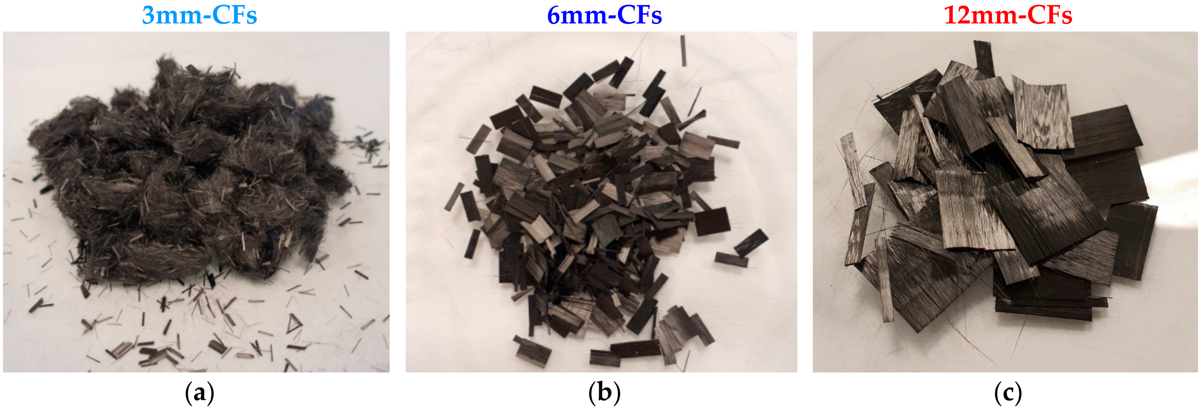

Figure S1. Bundles of carbon fibers: (**a**) 3 mm, (**b**) 6 mm and (**c**) 12 mm supplied by Apply Carbon.

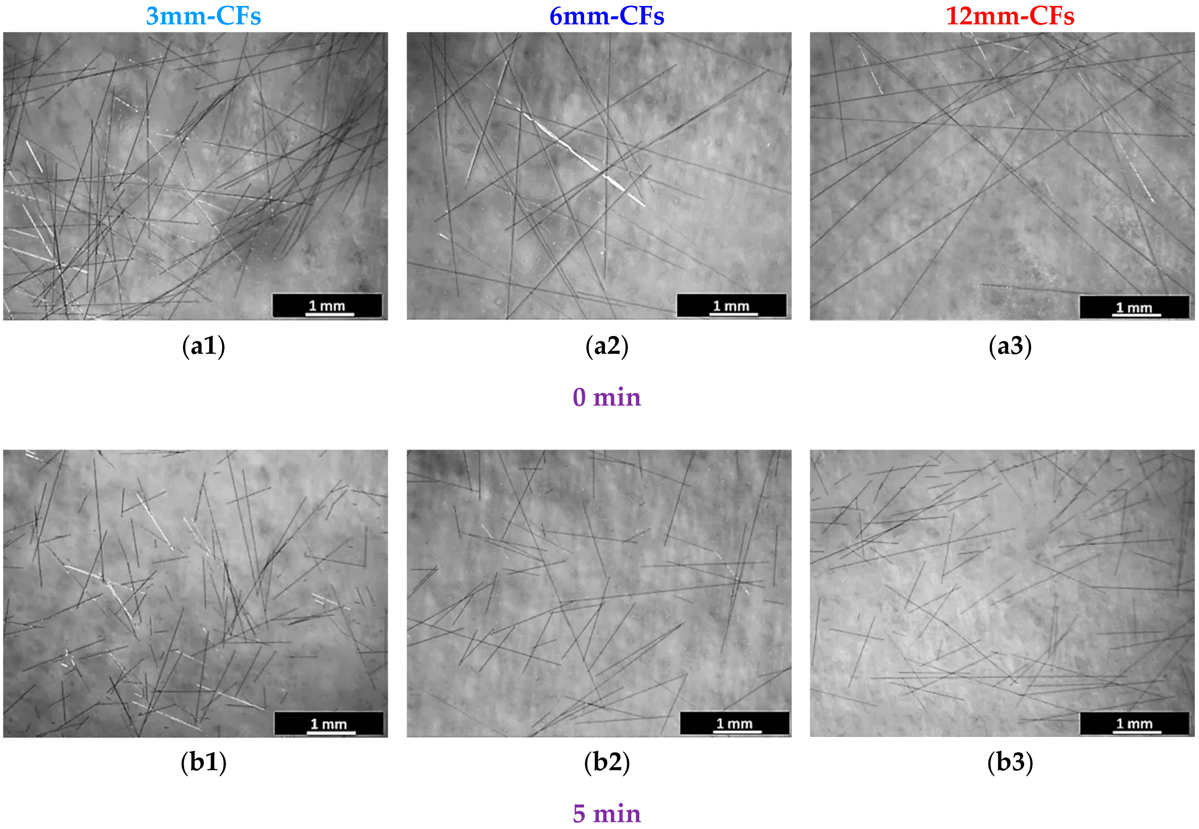

Figure S2. Microscopic images of (1) 3mm-CFs (2) 6mm-CFs (3) 12mm-CFs mixed with shear mixing during (**a**) 0 min (spatula) and (**b**) 5 min.

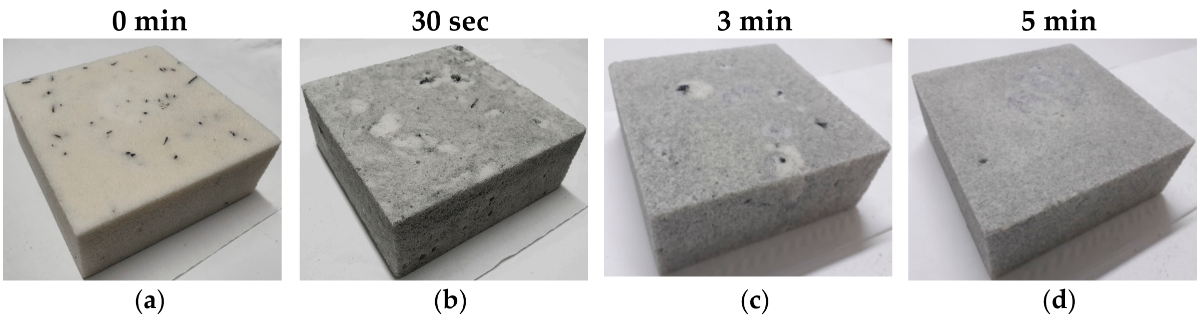

Figure S3. Epoxy foam loaded with 0.5 wt.% of 3mm-CFs and 6mm-CFs prepared with shear mixing for (**a**) 0 min, (**b**) 30 sec (**b**) 3 min and (**d**) 5 min.

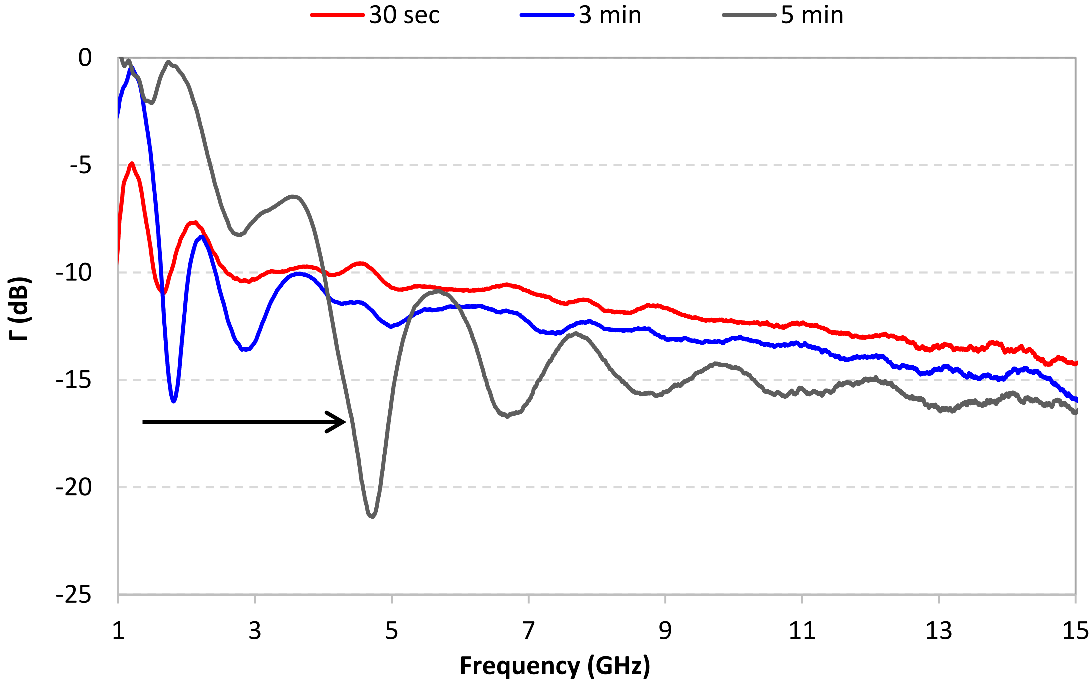

Figure S4. Frequency dependence of the reflection coefficient for epoxy foams loaded with 0.5 wt.% of 12mm-CFs for three times of shear mixing: 30 sec, 3 min and 5 min.

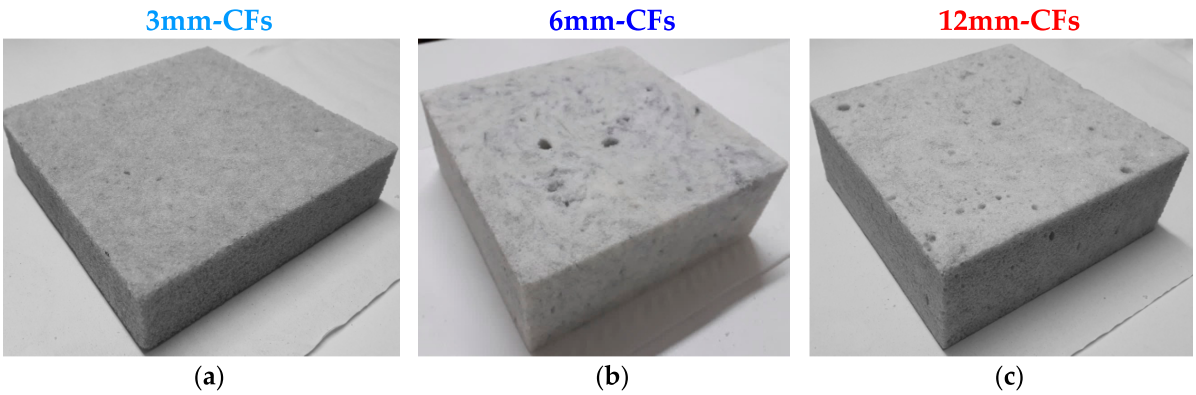

Figure S5. Photos of epoxy foams loaded with 0.25 wt.% of carbon fiber with length of (**a**) 3 mm (**b**) 6 mm and (**c**) 12 mm, elaborated using ultrasounds, during 5 min, for CFs dispersion.

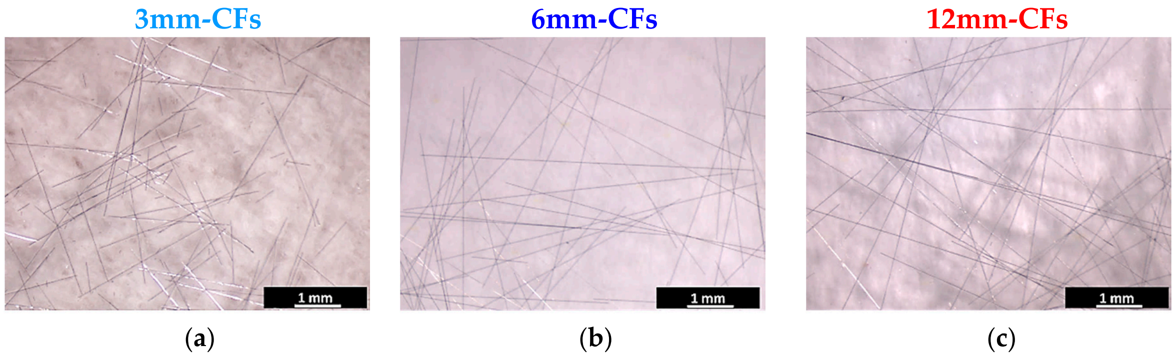

Figure S6. Microscopic images of (**a**) 3mm-CFs, (**b**) 6mm-CFs and (**c**) 12mm-CFs mixed with ultrasounds during 5 min.

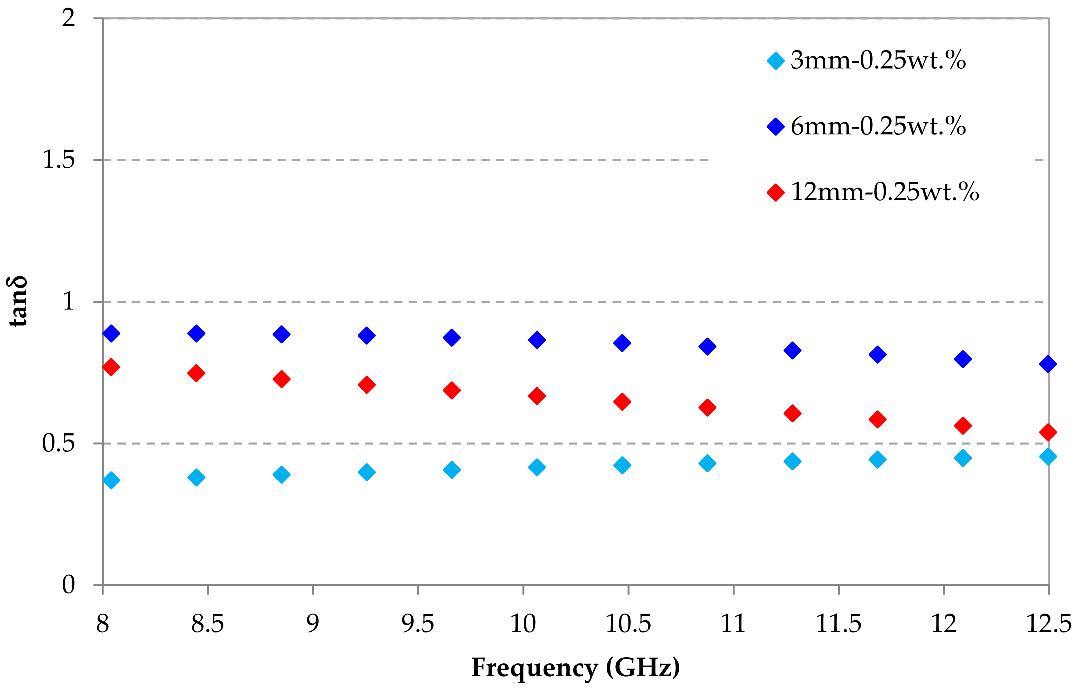

Figure S7. Dielectric losses of epoxy foams loaded with 0.25 wt.% of 3, 6 and 12mm-CFs, presented in X-Band frequency range.

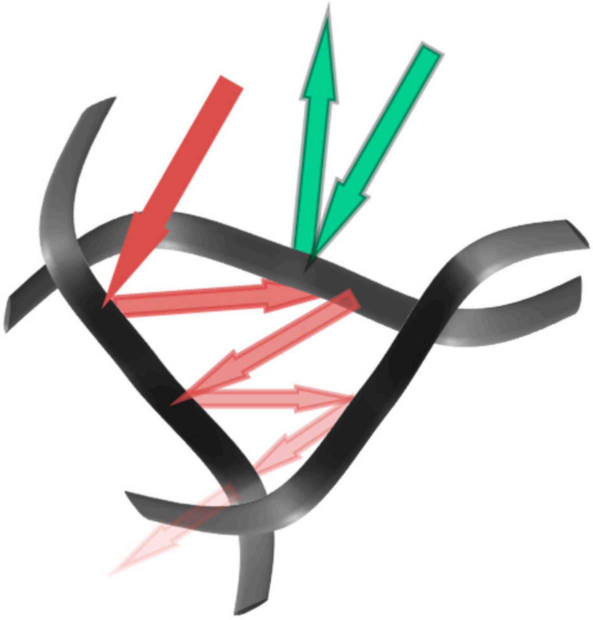

a) between long wavelength and crossed long CFs

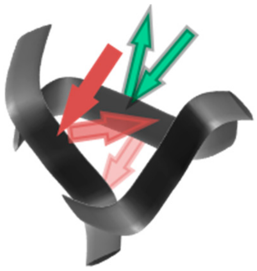

c) between short wavelength and crossed short CFs

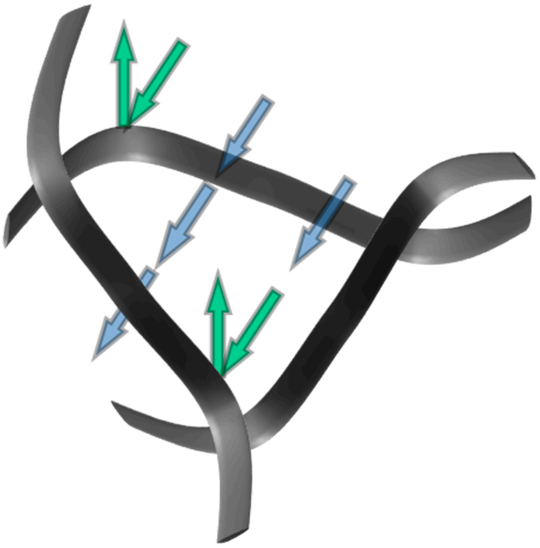

b) between short wavelength and crossed long CFs

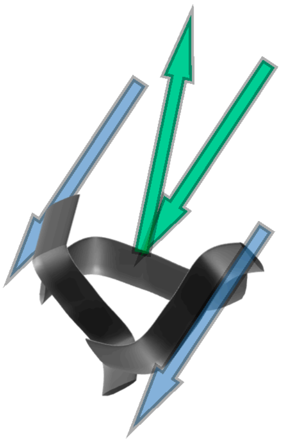

d) between long wavelength and crossed short CFs

Figure S8. Proposed multiple reflections and backscattering in CF agglomerates as a function of the CF length and EM wavelength. Trapped waves (Red arrows); transmitted waves (blue arrows); reflected waves (green arrows).

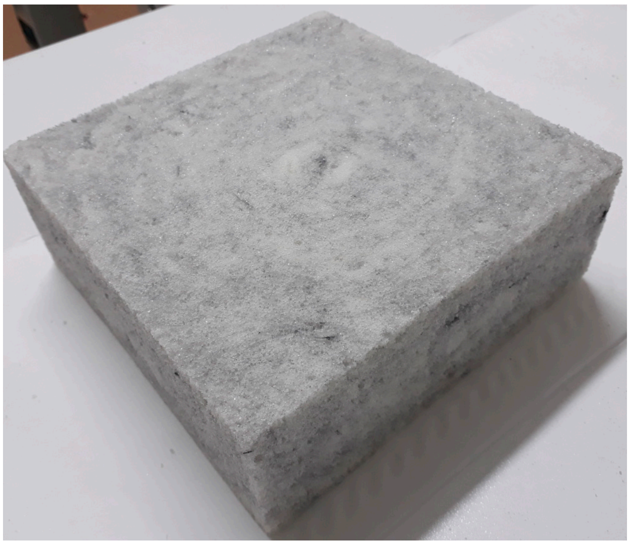

Figure S9. Photo of epoxy foams loaded with 0.25 wt.% of 6+12mm-CFs.

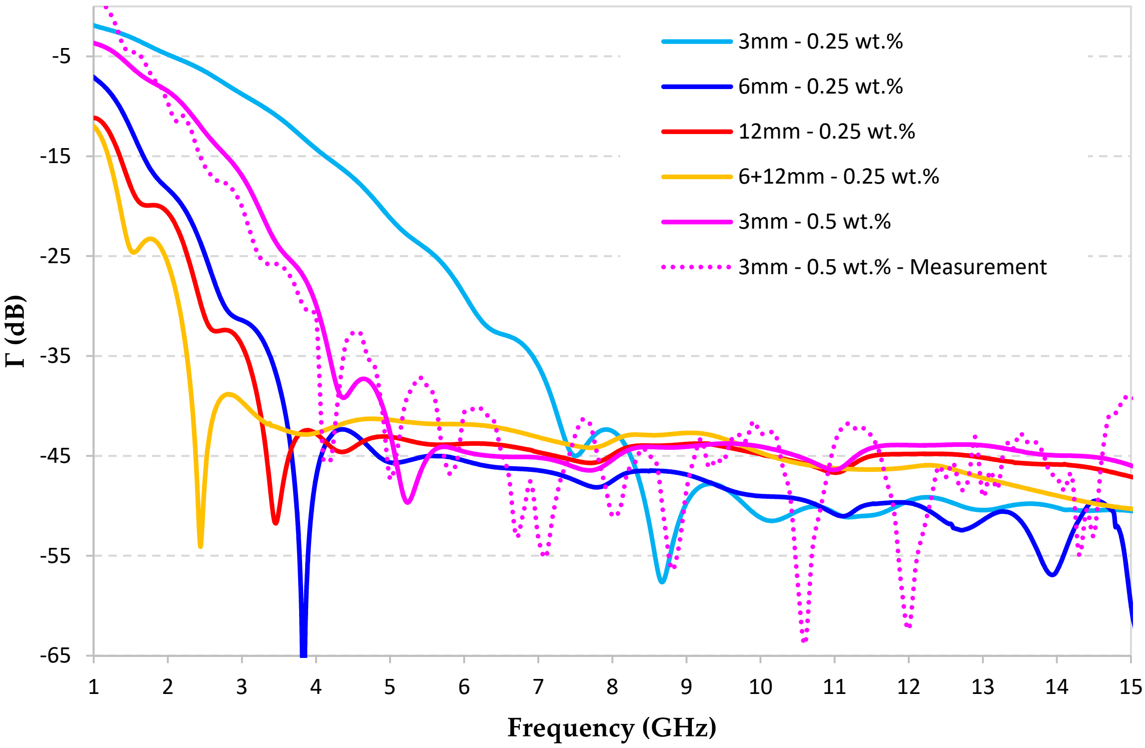

Figure S10. The frequency dependence of the simulated reflection coefficients for pyramidal epoxy foams loaded with 0.25 wt.% of 3, 6, 12 and 6+12mm-CFs compared to the measurement and the simulation of a prototype loaded with 0.5 wt.% of 3mm-CFs.
